# Characteristics and outcomes of cryptococcal meningitis in HIV seronegative children in Beijing, China, 2002–2013

**DOI:** 10.1186/s12879-016-1964-6

**Published:** 2016-11-04

**Authors:** Ling-yun Guo, Lin-lin Liu, Yue Liu, Tian-ming Chen, Shao-ying Li, Yong-hong Yang, Gang Liu

**Affiliations:** 1 Department of Infectious Diseases, Beijing Children’s Hospital, Capital Medical University, Beijing, China; 2 Key Laboratory of Major Diseases in Children and National Key Discipline of Pediatrics (Capital Medical University), Ministry of Education, National Clinical Research Centre for Respiratory Diseases, Beijing Key Laboratory of Pediatric Respiratory Infection Diseases, Beijing Pediatric Research Institute, Beijing Children’s Hospital, Capital Medical University, Beijing, China; 3 Department of Radiology, Beijing Children’s Hospital, Capital Medical University, Beijing, China

## Abstract

**Background:**

Data regarding HIV-seronegative pediatric patients with cryptococcal meningitis (CM) have been very limited.

**Methods:**

We retrospectively reviewed non-HIV-infected in patients with CM from January 2002 through December 2013 in Beijing Children’s Hospital. Records of the all patients were obtained and compared.

**Results:**

The 34 children had a median age of 5.6 years. Most of the patients were male (67.6 %). Only 23.5 % of the cases had identifiable underlying diseases. The sensitivity of the CSF cryptococcal antigen, India ink smear and CSF culture in our study were 81.5, 85.3 and 82.4 %, respectively. And the sensitivity of combinations of these tests was 91.2 %. Out of the 34 patients, 16 (47.1 %) had other organs involvement in addition to the brain. The main abnormal features via magnetic resonance imaging (MRI) were Virchow-Robin space dilatation (44.4 %), hydrocephalus (38.9 %), gelatinous pseudocysts (33.3 %), brain atrophy (33.3 %), meningeal enhancement (27.8 %) and local lesions (27.8 %). In total, 64.7 % of the patients were successfully treated at discharge, whereas treatment failed in 35.3 % of the patients.

**Conclusions:**

Cryptococcal meningitis is an infrequent disease with a high fatality rate in children in China. The majority of patients were apparently healthy. Clinicians should consider cryptococcal infection as a potential pathogen of pediatric meningitis. Cryptococcal antigen, India ink smear and culture tests are recommended for diagnosis.

## Background

Cryptococcal meningitis (CM) is the most common fungal infection of the central nervous system and has a high morbidity and mortality. Globally, approximately 957,900 cases of CM occur annually, resulting in 624,700 deaths within 3 months after infection [1]. Many large studies have been conducted in adults, but few studies have been dedicated to pediatric patients. On the basis of these limited data, the incidence of cryptococcosis in children was 0.016–100 cases/100,000 children [2]. The average annual incidence of CM was found to be 0.43/100,000 in China [3]. Reported pediatric patients only account for 2 % of all cryptococcal cases [4] but could potentially result in higher morbidity and mortality [5]. Furthermore, the average annual incidence of CM is less frequent especially in non-HIV (human immunodeficiency virus) pediatric population [1–3, 6–9]. Human immunodeficiency virus (HIV) infection is a main risk factor and accounts for 95 % of CM cases in middle- and low-income countries and 80 % of CM cases in high-income countries [7, 8]. However, the majority of adult patients with CM in China are apparently healthy (66.9 %) [9].

Data regarding predisposed and normal in children have been very limited. Here, we present a single-center study of CM in non-HIV-infected pediatric patients with a comprehensive analysis of clinical features and outcomes.

## Methods

### Study population

We retrospectively reviewed non-HIV-infected in patients with CM from January 2002 through December 2013 in Beijing Children’s Hospital (a 970-bed tertiary health care hospital), China. Records of all of the patients with CM during a 12-year period were obtained. Demographic data, underlying diseases, clinical features in the patients’ history, laboratory findings, cranial imaging, antifungal treatments and outcomes were analyzed.

### Definitions

#### Diagnosis of cryptococcal meningitis

A definite diagnosis of CM was determined if the patient met at least one of the following criteria [10]; (1) positive culture of *Cryptococcus* from cerebrospinal fluid (CSF), (2) positive India ink smear of CSF centrifuged sediment for *Cryptococcus,* or (3) positive cryptococcal antigen findings in the CSF and/or in the blood.

### Cryptococcal antigen assay

The Immy Latex-Crypto Antigens (Immuno-Mycologics, Inc.) were used to perform the antigen assay.

### Normal and predisposed hosts

Patients with one or more predisposing factors, such as underlying diseases (nephrotic syndrome, Henoch-Schonleinpurpura, juvenile rheumatoid arthritis, pyothorax, lung tuberculosis, X-linked agammaglobulinemia (XLA), hepatitis B virus infection, or congenital heart disease), were classified as predisposed hosts. Patients with no identifiable underlying diseases were classified as previously healthy hosts.

### The severity of fever

A low degree of fever indicates a temperature between 37.4 and 38 °C; a moderate degree of fever indicates a temperature between 38 and 39 °C; and a high degree of fever indicates a temperature more than 39 °C.

### Outcomes

Major outcomes could be in-hospital all-cause mortality and mortality at 6-months; and minor outcomes are CSF sterilization rate after the induction phase, the proportion of neurologicalsequelae (hearing and visual impairment) after the onset of disease and the length of hospitalization. Long-term outcomes were assessed via telephone at the end point of follow-up.

### Statistical analysis

Mean and standard deviations (SDs) are shown when distributions were confirmed normal; median and interquartile range (IQRs) are reported otherwise. The categorical variables were compared using the Chi-square test or Fischer’s exact test, as appropriate. Continuous variables within two groups were compared using the independent t-test for parametric data and the Mann-Whitney U test for non-parametric data. *P* values <0.05 were considered statistically significant. All of the statistical analyses were conducted using SPSS 17.0 (SPSS Inc., USA).

## Results

Overall, 34 HIV-negative children with CM were enrolled in this study.

### Baseline characteristics of the patients

**Fig. 1:**
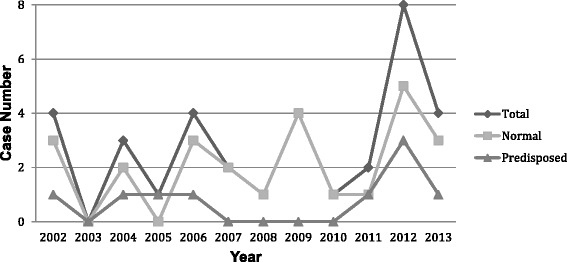
Number of cryptococcal meningitis cases diagnosed per year. The number of cases diagnosed remained constant each year except for 2012

The median onset age was 5.6 (range 1.6, 12.6) years and 88.2 % of the children were more than three years old. Twenty-three patients were male (67.6 %) and from a rural area (76.5 %). The male/female ratio was 2.09:1. Half of the patients (50 %) obtained the disease in the summer (June-August) during the 12-year period. Nearly half of the patients (44.1 %) had contact history with birds before the onset of illness. All of the children were Han people. The patients were from 10 provinces and districts of China; the largest subset of the population came from Hebei province. The numbers of cases were evenly distributed over the 12-year period with four cases every year apart from 2012 when there were eight cases (Fig. 1).

The median time from symptom onset to diagnosis was 30 (interquartile range 17, 54) days. In six cases (17.6 %), symptoms appeared less than two weeks before the diagnosis of CM, whereas in 23 patients (67.6 %), symptoms appeared between two weeks to two months prior to diagnosis. The other five patients (14.7 %) displayed symptoms more than two months prior to diagnosis. Of the participants, 70.6 % (24 cases) of the children were misdiagnosed as having other diseases before admission to our study, with tuberculosis (11/24, 45.8 %) accounting for the majority of these other diagnoses.

### Predisposing factors

All of the children were confirmed to be non-HIV infected by negative serum HIV antibodies. Only 8 cases (23.5 %) had identifiable underlying diseases. The following immunocompromising conditions were found in one of each of those eight cases: nephrotic syndrome, Henoch-Schonleinpurpura, juvenile rheumatoid arthritis, pyothorax, lung tuberculosis, X-linked agammaglobulinemia (XLA), hepatitis B virus infection, or congenital heart disease. The remaining 26 (76.5 %) patients were apparently healthy with basically normal levels of immunoglobulins and T cell subsets (CD4+ Tcells and CD8+ T cells). The CD4+ T lymphocyte count of all the patients were more than 500/mm^3^.

### Signs and symptoms

All of the patients had fever, which was the most common symptom, with 5.9 % (2 cases) presenting a low degree, 35.3 % (12 cases) presenting a moderate degree, and 58.8 % (20 cases) presenting a high degree. Other common clinical manifestations included nausea (22 cases, 64.7 %), headache (21 cases, 61.8 %), vomiting (21 cases, 61.8 %), meningeal signs (18 cases, 52.9 %) and neck stiffness (16 cases, 47.1 %). Eleven patients (32.4 %) presented with altered mental status, consciousness or fundus changes. Cranial nerve palsies were observed in 10 (29.4 %) cases involving the optic nerve, oculomotor nerve and facial nerve. Ten cases (29.4 %) experienced seizures, including five patients who had grand mal seizures. Five patients (14.7 %) presented limb weakness.

### Laboratory data

#### Blood findings

The median white blood cell (WBC) count in the blood was 10.5 (interquartile range 8.6, 14.9) × 10^9^/L with a predominance of neutrophils. 52.2 % of the patients had eosinophilia with the median eosinophil 1.07 (interquartile range 0.3, 1.24) × 10^9^/L. The median CRP was 15.6 mg/L with a maximum of 150 mg/L (inflammation was evaluated with a test that measured levels in excess of 8 mg/L). The erythrocyte sedimentation rate (ESR) was 46.5 ± 30.2 mm/h, and 18/29 (62.1 %) of the patients had elevated IgE levels. There were not any other clinical features of these in the children with eosinophilia and raised IgE, like recurrent boils, rashes etc., indicating that there was no suspected Hyperimmunoglobulin E Syndrome (Job’s syndrome) patients.

#### Cerebrospinal fluid (CSF) findings

All of the patients underwent lumbar puncture. 27/34 (79.4 %) had the medical records of CSF opening pressure .55.6 % (15/27) of the patients had high CSF opening pressure (>200 mmH2O) and the other 12 patients had normal CSF opening pressure. 8/15 (53.3 %) had the values records, 75 % (6/8) of the patients > 300 mmH_2_O, the other two patients’ values were between 200 ~ 300 mmH_2_O. The median WBC count in the CSF was 31 (interquartile range 9, 100) × 10^6^/L, with 67.6 % (23 cases) having pleocytosis. The median CSF glucose concentration was 2.38 ± 1.41 mmol/L, and 67.6 % (23 cases) had below normal levels (2.8 mmol/L). Seven of the 34 (20.6 %) patients had the ratio of CSF to plasma glucose at the meantime, and 42.9 % (3/7) of those patients had a CSF: glucose ratio that was less than 50 % of their plasma. The median CSF protein concentration was 585 (interquartile range 361, 860) mg/L, with 64.7 % (22 cases) having above normal levels (450 mg/L).

### Neuroradiological manifestation

Eighteen and 17 cases underwent cranial MRI or CT scans, respectively. All of the patients had abnormal results. Among these, the main radiological features via MRI were Virchow-Robin space dilatation (8/18, 44.4 %), hydrocephalus (7/18, 38.9 %), gelatinous pseudocysts (6/18, 33.3 %), brain atrophy (6/18, 33.3 %), meningeal enhancement (5/18, 27.8 %) and local lesions (5/18, 27.8 %). Common sites of Virchow-Robin space dilatation and gelatinous pseudocysts were the basal ganglia region and corona zone. The MRI scans also detected two cases of granuloma. We found that 90 % of the patients (9/10) had abnormal results as detected by magnetic resonance venography (MRV), mostly as transverse sinus and sigmoid sinus slim or discontinuous imaging. The vast majority of the patients obtained normal magnetic resonance angiography (MRA) results, whereas only two cases had a thinner middle cerebral artery. In contrast to MRI, common abnormal results for CT scans were hydrocephalus (9/17, 52.9 %) and brain atrophy (11/17, 64.7 %) without Virchow-Robin space dilatation and gelatinous pseudocysts.

### Electroencephalogram

In 12 cases, the patients underwent an electroencephalogram test, and seven cases (58.3 %) had abnormal results with varying slow wave levels.

### Pathogen findings

The sensitivity of the CSF cryptococcal antigen, India ink smear and CSF culture in our study were 81.5, 85.3 and 82.4 %, respectively. And the sensitivity of combinations of these three tests was 91.2 %. Of these, 88.2 % (30/34) of cases obtained positive results in the first three lumbar punctures. One patient obtained positive results in the ninth lumbar punctures. We had three patients who had all negative results of these three pathogen tests in the CSF, but had positive results antigens for Cryptococcus in the blood (Table 1).

**Table 1:** Pathogen findings of cryptococcal meningitis in CSF

Times	Antigen (%) (*n* = 27)	India ink smear (%) (*N* = 34)	Culture (%) (*N* = 34)	Combination (%) (*N* = 34)
1^st^	20 (74.1)	9 (26.5)	11 (32.4)	26 (76.5)
2^nd^	22 (81.5)	20 (58.9)	16 (47.1)	29 (85.3)
3^rd^	22 (81.5)	23 (67.6)	22 (64.7)	30 (88.2)
>3^rd^	22 (81.5)	29 (85.3)	28 (82.4)	31 (91.2)
Negative	5 (18.5)	5 (14.7)	6 (17.6)	3 (8.8)
Total	27 (100)	34 (100)	34 (100)	34 (100)

### Other organs involvement

Sixteen of the 34 patients (47.1 %) had other organs involvement in addition to the brain. All but one patient had lung involvement. Other organs included the liver, spleen, lymph node, renal and bone marrow. Infections were diagnosed in two patients by mesentery lymph node histopathology and sputum sampling, respectively.

### Initial antifungal therapy and outcomes

Four initial antifungal regimens were prescribed to the patients. A combination of amphotericin B and fluconazole, with or without flucytosine for 12 cases accounted for the first regimen, followed by fluconazole with or without flucytosine for 10 cases, and amphotericin B for five cases. Seven more patients were administered other treatments including amphotericin B colloidal dispersion, itraconazole, voriconazole, and others. Amphotericin B was given intrathecally to 11 patients (32.4 %) during hospitalization. Ventricular drainage or lumbar drainage was performed in two cases (5.9 %). Three patients (8.8 %) were transferred to the pediatric intensive care unit because of disease deterioration; two of these patients experienced mechanical ventilation. Two cases (5.9 %) experienced cerebral herniation during the hospitalization, three cases (8.8 %) had renal injury and 16 cases (47.1 %) had hypokalemia. In those patients who took amphotericin B therapy, 85.7 % of patients had anemia, 44.4 % had elevated aminotransferase and 15.4 % had neutropenia. There was no patient died in the hospital. Of all of the patients, 22 (64.7 %) obtained a successful outcome at discharge; half of those patients had a complete response, and the other half had a partial response. However, 12 cases (35.3 %) experienced failure results, with six cases having stable or progressive status at the time of discharge. CSF sterilization rate after the induction phase was 13.6 % (3/22). Fifteen patients under went followed up for a median time of two years. Seven patients (46.7 %) had a poor outcome at the endpoint, five of these patients (33.3 %) died, and two (13.3 %) were left with an altered mental status and visual impairment. Eight patients (53.3 %) recovered with no complications. No patients progressed to epilepsy. Compared to the survival group and the death group, the patients in death group were more likely to have neck stiffness (100 vs. 30 %, *P* < 0.05). The patients in survival group had higher ESR and eosinophil than patients in death group. There were no significant differences in baseline characteristics such as age, area, contact history, time to diagnosis, hospitalization, daily cost and CSF tests (Table 2).

**Table 2:** Comparison between the survival group and the death group (*N* = 15)

	Survival group (%) (*n* = 10)	Death group (%) (*n* = 5)	*P*
Age (year)	6 (4–10)	8 (5–10)	0.624
Sex (Male:Female)	3:2	1:4	0.282
Area (rural)	6 (60)	5 (100)	0.231
Contacting history	5 (50)	2 (40)	1.000
Predisposed hosts	4 (40)	2 (40)	1.000
Time to diagnosis (day)	28 ± 13	24 ± 20	0.732
Hospitalization (day)	53 ± 28	25 ± 23	0.084
Daily cost (RMB)	1258 (685–2502)	1304 (1187–2321)	0.540
Manifestations			
Fever	39.3 (38.6–39.8)	38.5 (38.5–39.2)	0.156
Headache	5 (50)	4 (80)	0.580
Vomiting	6 (60)	5 (100)	0.231
Altered mental status	1 (10)	3 (60)	0.077
Seizure	3 (30)	4 (80)	0.119
Vision damage	3 (30)	2 (40)	1.000
Neck stiffness	3 (30)	5 (100)	0.026*
Fundus abnormality	2 (25)	2 (100)	0.133
CSF			
CSF WBC (×10^6^/L)	24.5 (7.5–91.0)	16.0 (2.0–69.0)	0.581
Glu (mmol/L)	2.82 (1.88–3.76)	2.80 (1.54–5.40)	0.903
Pro (mg/L)	523 (333–856)	361(170–1492)	0.806
Blood			
WBC (×10^9^/L)	10.4 (8.6–18.2)	10.7 (9.0–12.1)	0.806
CRP (mg/L)	29.8 (7.8–41.6)	11.9 (1.7–54.5)	0.315
ESR (mm/h)	51 (44–76)	24 (16–31)	0.042*
EO (×10^9^/L)	1.37 (0.55–2.81)	0.2 (0.07–1.05)	0.040*

*P < 0.05

### Comparison between normal and predisposed hosts

There were 26 (76.5 %) normal host patients and eight (23.5 %) cases in the predisposed host group. The patients in the two hosts groups showed no significant differences in baseline characteristics such as age, area, contact history, time to diagnosis, hospitalization, daily cost and prognosis both at discharge and follow-up. In terms of manifestations, compared to normal hosts, the patients who had underlying diseases were more prone to having an altered mental status (62.5 vs. 19.2 %, *P* < 0.05) and seizures (62.5 vs. 15.4 %, *P* < 0.05). The patients in the normal hosts group had a higher ESR than patients in the predisposed hosts group. Other routine CSF and chemistry tests as well as blood findings had similar results in the two host groups (Table 3).

**Table 3:** Comparison between the predisposed hosts and normal hosts (*N* = 34)

	Normal hosts (%) (*n* = 26)	Predisposed hosts (%) (*n* = 8)	*P*
Age (year)	5 (3–9)	7 (4–10)	0.900
Sex (Male: Female)	19:7	1:1	0.388
Area (rural)	21 (80.8)	5 (62.5)	0.355
Contacting history	10 (38.5)	5 (62.5)	0.417
Time to diagnosis (day)	30 (20–56)	25 (13–56)	0.759
Hospitalization (day)	41 ± 25	53 ± 44	0.352
Daily cost (RMB)	846 (567–1615)	1080 (621–1080)	0.138
Disseminated cryptococcosis	14 (53.8)	2 (25.0)	0.233
Manifestations			
Fever	39.2 ± 0.7	38.9 ± 0.8	0.242
Headache	14 (53.8)	7 (87.5)	0.116
Vomiting	14 (53.8)	7 (87.5)	0.116
Altered mental status	5 (19.2)	5 (62.5)	0.031*
Seizure	4 (15.4)	5 (62.5)	0.017*
Vision damage	4 (15.4)	4 (50.0)	0.066
Neck stiffness	13 (50.0)	5 (62.5)	0.693
Blood			
WBC (×10^9^/L)	12.4 (8.6–16.3)	10.2 (8.8–11.3)	0.274
CRP (mg/L)	11.9 (0–63.5)	16 (0–30.9)	0.592
ESR (mm/h)	50 (26–79)	10 (1–52)	0.037*
EO (×10^9^/L)	0.72 (0.31–1.77)	0.38 (0.2–1.24)	0.504
CSF			
WBC (×10^6^/L)	32 (10–113)	25 (7–102)	0.313
Glu (mmol/L)	2.24 ± 1.23	2.80 ± 1.92	0.332
Pro (mg/L)	625 (436–890)	660 (386–938)	0.528
Prognosis			
Success at discharge	16 (61.5)	6 (75.0)	0.618
Success at follow-up	4/10 (40.0)	3/5 (60.0)	0.608

*P < 0.05

## Discussion

The central nervous system is the main involved area in cryptococcosis disease both in pediatric and adults’ population. Data showed that there was 83.4 % of central nervous system involvement in 8769 cases of cryptococcosis in China from 1985 to 2010 [11] and 87.1 % of the pediatric cases had central nervous system involvement in China [4]. However, this is less frequent pediatric cases reported, particularly in non-HIV patients [1–3, 6–9]. Reported pediatric patients only account for 2 % of all cryptococcalcases [4] but could potentially result in higher morbidity and mortality rates [5].

In our study, 67.6 % of the patients were boys. This is similar to other hospital-based reports of CM in both adults and children with male predominance [2, 3, 12–17]. Why there is a male predominance in patients with CM is an interesting question requiring further evaluation. In our series, most of the patients were from rural areas with a history of contacting birds. Guo Jiahua reported that 73.9 and 73.26 % of infected children were from rural areas and had a history of avian contact in Shi Jiazhuang, China [3]. Similar results were also obtained in a population-based study in Australia and New Zealand [18]. The reason for this phenomenon is unknown but may partly be due to increased environmental exposure.

The median age of the infected children was approximately 5.6 years old in the current study, and only three cases were younger than two years of age consistent with prior studies of CM in non-HIV children [2, 3, 15, 18]. Only 3 % of the cases in the reported literature and 9.5 % of new patients are younger than 2 years [19]. This finding could be explained by the increase in the mobility and independence of children, resulting in increased environmental exposure.

In our study, 52.2 and 62.1 % of the patients had an eosinophilia or elevated IgE level, respectively. There were not any other clinical features of these in the children with eosinophilia and raised IgE, indicating that there was no suspected Hyperimmunoglobulin E Syndrome. Although eosinophilia has been reported in HIV-negative cryptococcal infection patients, it is still relatively rare [11, 20–23]. Yamaguchi reported a 23-year-old patient was diagnosed as disseminated cryptococcosis including meningitis with very marked eosinophilia (75 %). And the eosinophilia was successfully alleviated by treatment for cryptococcal meningitis [24]. In addition, infections presenting with elevated IgE have been associated with a high risk of systemic cryptococcal dissemination, including meningitis [25, 26]. Th_2_-dominant immune status has been reported to cause increases in serum IgE, as well as susceptibility to cryptococcal infections [27].

In terms of clinical symptoms and signs, in the current study, fever was the most frequent symptom, followed by nausea, vomiting, headaches, meningeal signs, neck stiffness, altered mental status and consciousness, seizure and cranial nerve palsies. Visual and hearing impairment were less common. These results were similar to those described in the literature [3, 9, 15, 18]. Pleocytosis, elevated CSF proteins, and decreased CSF glucose levels were also common. Such findings are also consistent with previous reports of cryptococcosis in non-HIV-infected children by Guo and Zhu [3, 15, 18].

Notably, 70.6 % of the children were diagnosed as having other diseases before admission in our study, with tuberculosis accounting for the most frequent misdiagnosis. It is unexpected that up to 70–100 % of CM patients experienced misdiagnosis in previous studies, mostly as tuberculosis-associated diseases [15]. CM is an important differential diagnosis with tuberculosis. Moreover, it takes a long time to diagnose this disease. Thus, the tests for pathogen detection play an important role in helping doctors make a definite diagnosis. The high sensitivity of combinations with variety cryptococcal tests in our study is encouraging. The sensitivity of the CSF cryptococcal antigen, India ink smear and CSF culture in our study were 81.5, 85.3 and 82.4 %, respectively. Apparently, the India ink stain, culture and cryptococcal antigen tests should be performed in children suspected to have CM, even those with normal cerebrospinal fluid and biochemical test results.

Consistent with other studies, CM patients had poor outcomes with a longer hospitalization duration despite multi-antifungal treatments [3, 15]. 35.2 % of the patients had no response at discharge and 33.3 % died during the follow-up time. Therefore, CM in pediatric populations remains a large challenge for pediatricians.

The proportion of immunocompetent patients with CM varies among non-HIV-infected patients in different regions and countries regardless of the age. Studies from the U.S., France, and Thailand reported a relatively small rate (17–35 %) of cryptococcosis occurring in patients with an apparently healthy condition [8, 14, 28]. However, a significantly higher rate of cryptococcosis was reported in immunocompetent individuals in China. In our study, only 8 cases (23.5 %) had identifiable underlying diseases, and the remaining 26 (76.5 %) patients were apparently healthy with basically normal levels of CDs and immunoglobulins. This is consistent with other studies. For example, among the 154 CM cases reported at Huashan Hospital, Shanghai, 66.9 % cases were identified in immunocompetent individuals [9]. Similarly, 68 % of CM cases were reported in immunocompetent individuals in another study performed at Changzheng Hospital, Shanghai [29]. In terms of the 23 HIV-negative children with CM in Shi Jiazhuang, China, only 26.1 % of the cases had underlying diseases. A population-based study reported that 61.9 % of cryptococcosis cases were from immunocompetent individuals in Taiwan [30]. Furthermore, studies from other Asian countries where Chinese is the predominant ethnic group reported similar results. In a study on HIV-uninfected patients with CM in Vietnam, 81 % (57 patients) had no clear predisposing factor [31]. Taken together, these reports suggest that cryptococcosis predominantly occurs in immunocompetent hosts in the Chinese ethnic population, which may be more susceptible to cryptococcal infections than other ethnic groups. The reason for this phenomenon is not well known. Ou et al. [32] performed a study regarding the association between mannose-binding lectin (MBL) polymorphisms and the development of CM in non-HIV Chinese patients. The authors found that the genotypes underlying deficient MBL production were associated with CM in the Chinese Han ethnicity, particularly in immunocompetent patients, suggesting that MBL deficiency is a genetic predisposition to CM. Another investigation revealed that FccRIIB polymorphisms are an important genetic factor contributing to CM in HIV-uninfected Chinese individuals [33].

To our knowledge, the present study is one of the largest case series of CM in non-HIV pediatric patients around world. However, this study has some limitations. It was a single center, hospital-based retrospective design, which introduces the possibility of unrecognized biases, a high dropout rate and incomplete data collection including CSF pressure measurements, EO etc. Because of cryptococcal meningitis in non-HIV pediatric population being relatively rare, we had a small sample in the study and the small number of deaths precluding multivariate analysis of factors influencing mortality. We had no data regarding pathogen serotype or molecular type. Thus, we would like to use our central nervous system follow-up system to perform more prospective studies in the future. Further multicenter studies are necessary to enrich the generalizability of CM in children.

## Conclusions

CM is an infrequent disease with a high fatality rate in children in China. The majority of patients were apparently healthy. Clinicians should consider cryptococcal infection as a potential pathogen of pediatric meningitis. Cryptococcal antigen, India ink smear and culture tests are recommended.

## Abbreviations

AmB: Amphotericin B deoxycholate
CM: Cryptococcal meningitis
CSF: Cerebrospinal fluid
ESR: Erythrocyte sedimentation rate
FC: Flucytosine
Flu: Fluconazole
HIV: Human immunodeficiency virus
MBL: Mannose-binding lectin
MRA: Magnetic resonance angiography
MRI: Magnetic resonance imaging
MRV: Magnetic resonance venography
WBC: White blood cell
XLA: X-linked agammaglobulinemia
